# Physical Treatment in Heart Disease

**Published:** 1896-02-08

**Authors:** Theodora Johnson

**Affiliations:** the Swedish Institute for Physical Training, Clifton.


### Physical Treatment in Heart Disease.

By Theodora Johnson, the Swedish Institute for Physical Training, Clifton.

Some aspects of the treatment of heart disease by specialised exercises, which have recently been discussed, are of such importance as to merit reconsideration.

In his article on this subject Dr. Wilde[1] associated with a Swedish exercise methods which are in one respect at least completely at variance with the fundamental principles on which Ling's treatment is based. Inasmuch as the resistance to the outflow of blood from the heart increases in direct ratio to the force of expiration, and consequently reaches the maximum when the respiration is absolutely checked by forcible closure of the glottis as when the breath is held during exercise. Ling insisted that it was most essential that the breathing should always be regular and continuous throughout the movements, and he pointed out that if respiration were checked, the holding of the breath threw undue strain upon the heart muscles, producing an effect directly opposite to that desired. Especially does this apply in the treatment of heart disease by exercise. Ling fully realised that it is "so natural to hold the breath during any special exertions that it becomes an involuntary and unconscious act," but considered that a fault to be carefully guarded against. We may further bear in mind that in performing movements for medical purposes it is not our object to obtain "the maximum of force with the minimum of strain."

There is probably no class of athletics which throws more excessive strain on the heart than race-rowing, and that it not infrequently leads to the development of various forms of heart disease is a well recognised fact. We therefore think that Dr. Wilde is unfortunate in taking the physiological respiratory conditions in rowing races as the type to be imitated in the treatment of cardiac affections. Surely respiration in race-rowing might be more fittingly cited as the antithesis of that suited to cardiac therapy.

The exercise which Dr. Wilde recommends, and describes in his final paragraph, is one of the Swedish movements, and is much used for its effects on the circulation and respiration, but with this essential difference, that the expiration takes place during the downward movement of the arms, instead of with the breath held until the termination of the movement, as given by him.

Dr. Wilde states that the movements described by Dr. Fisher[2] as the Nauheim movements are Ling's exercises. They are similar, but not identical. For instance, diagrams 1, 3, and 4 present a difference which was considered a distinct defect by Ling, and, therefore, carefully excluded from his exercises—the hands touch, and so tend to contract the chest; in Ling's exercises they are kept the shoulders' width apart. Diagrams 6, 12, 13, and 14 are also open to criticism were they given as Ling's exercises, though they may be correct as the Nauheim movements. As a Swedish exercise, figure 6 would have the hands fixed at the sides, the muscles of the back well contracted, the chest brought forward, and the head raised. In figure 12 the knee should be raised to a horizontal line with the hip, and the toe should be stretched downward. In figures 13 and 14 the foot would also be extended, causing greater muscular action.

We must further take exception to the statement that "instructors in the Swedish movements direct that the mouth should be kept open." Even and deep respiration through the nose that the air may be warmed and filtered in the nasal passages is the invariable rule in Ling's system.

[1] The Hospital, February 1st, 1896. [2] Ibid, January 11th, 1896.

